# Relationships among Hydrogen Peroxide Concentration, Catalase, Glucose Oxidase, and Antimicrobial Activities of Honeys

**DOI:** 10.3390/foods13091344

**Published:** 2024-04-26

**Authors:** Sandra M. Osés, Carlos Rodríguez, Olga Valencia, Miguel A. Fernández-Muiño, M. Teresa Sancho

**Affiliations:** 1 Department of Biotechnology and Food Science, Universidad de Burgos (University of Burgos), Pza. Misael Bañuelos s/n, 09001 Burgos, Spain; car.crg@gmail.com (C.R.); mafernan@ubu.es (M.A.F.-M.); mtsancho@ubu.es (M.T.S.); 2 Department of Mathematics and Computation, Universidad de Burgos (University of Burgos), Pza. Misael Bañuelos s/n, 09001 Burgos, Spain; oval@ubu.es

**Keywords:** honey, hydrogen peroxide, glucose oxidase, catalase, antimicrobial activity

## Abstract

Honey is a natural sweetener made by bees that exhibits antimicrobial activity, mainly related to its H_2_O_2_ content. The aim of this work was to research the H_2_O_2_ concentration of 24 Spanish honeys from different botanical origins, studying their possible correlation with glucose oxidase (GOx), catalase (CAT), and anti-*Staphylococcus aureus* activities (minimal inhibition concentration (MIC), minimal bactericidal concentration (MBC), and percentage of inhibition at 5% (*w*/*v*) honey against *Staphylococcus aureus*), as well as possible correlations among all the analyzed parameters. The results showed that the H_2_O_2_ concentration did not depend on the botanical origin of the honeys. There were neither correlations between the H_2_O_2_ concentration and the activities of GOx and CAT, nor between GOx and antimicrobial activity. However, CAT and antimicrobial activities were positively correlated. Therefore, CAT could be successfully used as a possible marker of the antimicrobial activity of honeys against *Staphylococcus aureus*. Furthermore, a linear regression model has been fitted to explain the antimicrobial activity from CAT and GOx activity and H_2_O_2_ concentration. Although H_2_O_2_ is one of the compounds involved in honey’s antibacterial activity, this capacity also strongly depends on other honey components (such as low water activity, acidity, osmolarity, and phenolic compounds). The very high anti-*Staphylococcus aureus* activity exhibited by all samples could be interesting for commercial honey-based formulations also helping to promote local beekeeping.

## 1. Introduction

*Staphylococcus aureus* is one of the bacteria often associated with wound and burn infections [1], being on the World Health Organization’s list of priority pathogens for which antibiotics are highly needed [2]. Nowadays, due to the increase in multi-drug resistance organisms, researchers are looking for several alternatives.

Honey is a natural and traditional food made by bees from flowers’ nectar or honeydew, which is collected, processed, and stored in hives. This product has been used since ancient times (6000 BC) as sweetener for humans [3], exhibiting antioxidant, antibacterial, and anti-inflammatory properties. Therefore, honey has been used for medical purposes [4], such as the treatment of surface wounds, burns, and inflammation in natural medicine [5,6,7]. Commercial honey-based formulations (gels, dressing, ointment, cream, paste, syrup, and pastilles, among many others) are nowadays widely available on the market [8]. Most of these products contain Manuka honey or buckwheat honey, with sage and honeydew honeys also being used as ingredients. Research on the antibacterial activities and antibacterial-related parameters of other honeys from different botanical origins could open different commercialization possibilities, helping boost local beekeeping practices.

Honey exhibits antimicrobial activity against different bacteria, fungi, viruses, and parasites [9]. The antimicrobial activity of honey has been commonly divided into peroxide (hydrogen peroxide) and non-peroxide factors. Non-peroxide factors include low water activity, acidity, osmolarity, phenolic compounds, methylglyoxal (Manuka honey), defensin-1, lysozyme, volatile compounds, and lactic bacteria [9,10,11]. H_2_O_2_ is a honey compound responsible for most honey bacteriostatic and bactericidal activity [12,13,14,15]. The hydrogen peroxide concentration in a given honey depends on its glucose oxidase and catalase activities [14]. Hydrogen peroxide is produced from glucose (glucose + water + oxygen → gluconic acid + H_2_O_2_) by the enzyme glucose oxidase (GOx) that is synthesized in the hypopharyngeal glands of honeybees [16]. Catalase (CAT) originates from pollen, being mainly responsible for hydrolyzing H_2_O_2_ to water and oxygen. GOx and CAT are of utmost importance for honey quality control, being positively or negatively related to antioxidant and antimicrobial activities. However, the analysis of both enzymes is complex, taking a considerably longer time than the analysis of other honey enzymes, such as diastase or invertase. GOx has been analyzed in several studies. In contrast, CAT has not been so extensively researched, although Weston [14] described the important role that this enzyme has in the antimicrobial activity of honeys.

Different studies have concluded that despite there being a correlation between the antimicrobial activity of honey and its H_2_O_2_ concentration, there is no relation between H_2_O_2_ and GOx due to the complex pathways of H_2_O_2_ production and destruction. Brudzynski [17] reviewed the relation of the H_2_O_2_ variation with different factors, such as the different GOx bee production (related to age and cast of the bee and pollen nutrition), glucose concentration, water activity, and osmolarity (high glucose concentration and osmolarity and low water activity reduced the mobility of molecules, inhibiting the GOx reaction), H_2_O_2_ accumulation, sensitivity of H_2_O_2_ to light and heat, catalase activity, decomposition by metal-containing enzymes and ascorbic acid, products from polyphenols autoxidation, presence of GOx in some flowers, or/and H_2_O_2_ production by microorganisms present in honey [18,19,20]. Godocikova [13] highlighted the importance of studying the relation between GOx and CAT activities, because these two enzymes had not been broadly analyzed in different commercial honeys at the same time yet. Sagona [21] verified that both enzymes together with other factors such as gluconic acid and phenolic acid seemed to play a role in the microbial inhibitory activity of honeys.

After China, the European Union (EU) is the world’s second-largest honey producer. Although the total number of beehives is increasing, the EU is only 60% self-sufficient with regard to honey, so imports are needed to cover the EU’s domestic consumption [22]. The quality and properties of honeys are key purchasing factors for consumers and users. Therefore, simplifying the analytical procedures related to beneficial properties that would be written on the honey label is of paramount importance nowadays.

Possible relationships between the antimicrobial activity of honeys, concentration of H_2_O_2_, as well as GOx and CAT activities have hardly been researched. GOx is naturally present in an inactive state in honey due to the low pH conditions. When honey is diluted, GOx is activated [11]. Thus, we consider that it is of utmost importance to determine the real activity of GOx instead of the GOx content that was already measured by other researchers [13,15,23]. Therefore, the purpose of this research was to determine the possible relationships among CAT and GOx activities, the concentration of H_2_O_2_, and the antimicrobial activity of honeys against *Staphylococcus aureus*.

## 2. Materials and Methods

### 2.1. Materials

Reagents: Sodium dihydrogen phosphate (99.99%) and di-sodium hydrogen phosphate anhydrous (99.99%) were acquired from Merck (Steinheim, Germany). *o*-dianisidine dihydrochloride (D9154), horseradish peroxidase (P8375-5KU), hydrogen peroxide (>30%), and dialysis membrane (D6191) were acquired from Sigma-Aldrich (St. Louis, MO, USA), which is a part of Merck. Potassium permanganate, sodium oxalate, hydrochloric acid, Baird Parker agar base (BP), and egg yolk emulsion with potassium tellurite were acquired from VWR International Eurolab, which is part of Avantor (Llinars del Vallés, Cataluña, Spain). D-(+)-Glucose (99.99%) was acquired from Panreac (Barcelona, Spain). Nutrient broth no. 2 (NB) and Ringer’s solution were acquired from Oxoid, which is part of Thermo Fisher (Basingstoke, Hampshire, UK). Water was deionized using a Milli-Q water purification system (Wasserlab, Navarra, Spain).

Apparatus: We used the Varian Cary Bio 400 spectrophotometer (Varian, part of Agilent Technologies, Santa Clara, CA, USA) and fluorometer Varioskan LUX microplate reader (Thermo Fisher, Kanderl, Germany). We also used sterile 96-well round-bottomed polystyrene microtiter plates (Brand, Wertheim, Germany).

### 2.2. Honey Samples

This study was carried out with twenty-four honeys from different botanical origins harvested in 2020 by beekeepers from Castilla y León (Spain). Botanical origins (Table 1) were determined using melissopalynology and sensory analyses [24,25,26,27,28,29]. The samples were stored in the dark at room temperature (20 °C ± 2 °C) until the analyses (for 5 months).

### 2.3. Catalase and Glucose Oxidase Activity

Sample dialysis: Honey (7.5 g) was dissolved in 4 mL of 0.015 M phosphate buffer (pH 7.0) and quantitatively transferred to the previously activated dialysis membrane. The membrane was closed with tubing clasps and placed into a beaker containing 3 L of phosphate buffer 0.015 M and stored at 4 °C for 11 h. Then, the membrane was placed into another 3 L beaker containing fresh 0.015 M phosphate buffer and stored for another 11 h. Finally, the honey was transferred to a 50 mL volumetric flask at room temperature, and we rinsed the membrane with buffer and used rinsing to dilute the dialyzed honey to the volume [30]. This sample was used for the analysis of GOx and CAT activities. 

Catalase activity was performed by the procedure described by Huidobro et al. [30]. The procedure was based on the reaction between the remaining H_2_O_2_, after honey’s catalase, and *o*-dianisidine and peroxidase, measuring the absorbance of the colored product at 400 nm during 30 min at 5, 10, 20, and 30 min each. The result was expressed as the catalase activity (Kf: min^−1^ g^−1^) per gram of honey. The standard curve was prepared from 0.00 to 22.48 µg H_2_O_2_. 

Glucose oxidase activity was performed by the procedure described by Sánchez-Castro [31]. The procedure was based on the reaction between the H_2_O_2_ formed in the GOx reaction with *o*-dianisidine and peroxidase, measuring the colored product spectrophotometrically at 400 nm. The glucose oxidase activity was calculated as the amount of H_2_O_2_ (µg) that is obtained in 1 h through the effect of the glucose oxidase contained in 1 g of honey (µg H_2_O_2_ h^−1^ g^−1^) [18]. The standard curve was prepared from 0.00 to 10.11 µg H_2_O_2_. 

### 2.4. Hydrogen Peroxide Concentration

The hydrogen peroxide concentration was performed according to the manufacturer’s instruction of the Flourimetric Hydrogen Peroxide Assay Kit (MAK 165, Sigma-Aldrich). The fluorescence formed was measured at 540 nm excitation and at 590 nm emission after 15 min of incubation at room temperature. The standard curve was generated using dilutions of a fresh 20 mM H_2_O_2_ stock solution.

### 2.5. Antimicrobial Activity

The antimicrobial activity of honeys was assayed against *Staphylococcus aureus* CECT 435. Bacterium was grown in BP with egg yolk with potassium tellurite for 24 h at 37 °C. Then, one colony was grown in NB 18 h at 37 °C. Cell suspensions were diluted in sterile Ringer’s solution to obtain initial cell counts of 6 log cfu/mL (determined using plate counts).

The antimicrobial activity was evaluated by a broth microdilution assay. For each sample, 9 different honey concentrations (60%, 40%, 30%, 20%, 15%, 10%, 5%, 1%, and 0.5%) dissolved in NB were studied. A total of 80 µL of 6 log CFU/mL of *S. aureus* was mixed with 720 µL of each sample dilution in sterile Eppendorf tubes. Finally, 200 µL of each tube was pipetted by triplicate into the wells, incubating the plate for 24 h at 37 °C. The contamination control (180 µL honey dilution + 20 µL Ringer), positive control (180 µL NB + 20 µL *S. aureus*), and negative control (170 µL NB + 20 µL *S. aureus* + 10 µL bleach) were also established. The minimum inhibitory concentration was defined as the minimum honey concentration where no turbidity was observed after incubation for 24 h at 37 °C. Then, 10 µL of each well, in which no turbidity was observed, was plated on BP and incubated for 24 h at 37 °C. The minimum bactericidal concentration was defined as the minimum concentration of honey at which bacterium did not grow on the agar plates.

Furthermore, the optical density of each well was measured at 600 nm before and after incubation to determine *S. aureus* growth and the honeys’ inhibition percentage. The inhibition percentage at a 5% honey concentration was calculated considering 100% of growth of the subtraction of the optical density of the wells containing 200 µL of sterile NB from the optical density of the positive control. 

### 2.6. Statistical Analysis

All the analyses were performed in triplicate, expressing the results as mean values and standard deviations. A one-way analysis of variance (ANOVA) followed by the LSD honestly significant difference test (*p* < 0.05) were performed. Pearson correlations were applied to the results. A multiple regression model was fitted to explain the inhibition percentage at a 5% honey concentration, from CAT and GOx activity, and the H_2_O_2_ concentration. The statistical software Statgraphics Centurion XIX was used (Statgraphics Technologies, Inc., The Plains, VA, USA).

## 3. Results and Discussion

### 3.1. Catalase and Glucose Oxidase Activity

**Figure 1:**
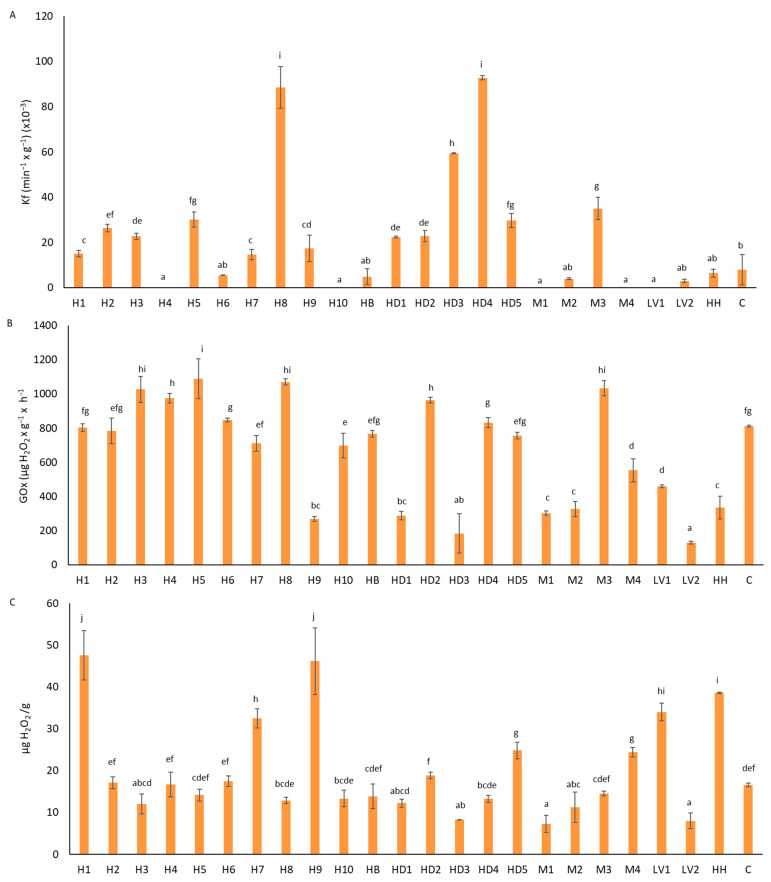
Catalase activity (**A**), glucose oxidase activity (**B**), and H_2_O_2_ concentration (**C**) in Spanish honeys from different botanical origins. H: ling heather; HB: ling heather and broom; HD: honeydew; M: multifloral; LV: lavender; HH: holly; C: cornflower. a–j: different letters indicate significant differences (*p* < 0.05) between honey samples. (*n* = 3).

The honeys showed mean values of catalase activity ranging between non-detected and 88.54 × 10^−3^ min^−1^ g^−1^ (Figure 1A). These values were similar to the results obtained by other authors [21,30,32]. In the honeys in which catalase was not detected, diastase and hydroxymethylfurfural (HMF) were determined. Assays of diastase and HMF were performed according to the harmonized methods of the International Honey Commission [33]. The results of both parameters were in accordance with the standards [34,35], discarding the idea that long storage could be the cause of the non-detection of catalase activity. The CAT activity was very variable. In general, honeydew honeys exhibited the highest activities, while lavender honeys showed the lowest ones. Catalase is the main factor responsible for H_2_O_2_ degradation, and its origin is vegetal. Therefore, the catalase activity varied depending on the botanical origin of the samples. Other researchers showed that heather (*Erica* spp.) and honeydew honeys had higher catalase activity than other honeys [30]. 

The mean GOx activity for each honey ranged between 130 and 1089 µg H_2_O_2_ g^−1^ h^−1^ (Figure 1B). The GOx activities in our Spanish honeys were higher that the activities described by other authors in Croatian (25–400 µg H_2_O_2_ g^−1^ h^−1^) and Turkish honeys (0 and 11.2 µg H_2_O_2_ g^−1^ h^−1^) [36,37,38], but similar to those obtained by Sánchez [31] in other Spanish honeys (169–858 µg H_2_O_2_ g^−1^ h^−1^). These discrepancies can be explained considering that the GOx activity significantly varies in different honeys due to various factors, such as pollen in the apiary, bee’s age, bee’s tasks, and the genetic diversity of honeybees [20]. Although the botanical origin could have little to do with the GOx activity [20], our results showed that heather honeys in general had higher activity and lavender honeys had lower activity than others. Strelec et al. [36] and Flanjak et al. [37] described significant differences according to botanical origin, exhibiting that black locust honeys had a considerably lower GOx activity than sage, chestnut, lime, mint, and honeydew honeys.

### 3.2. Hydrogen Peroxide Concentration

The H_2_O_2_ concentration in honeys ranged from 7.26 to 47.56 µg H_2_O_2_ g^−1^ (Figure 1C). These data agree with the values obtained by other authors, ranging from 1 to 47.2 µg g^−1^ [15,39,40,41]. We did not find any relationship between the amount of H_2_O_2_ and the botanical origin of honeys, unlike other researchers [42] who described that honeydew and chestnut honey contained large amounts of H_2_O_2_, while acacia, heather, and rape honeys had a lower capacity to produce hydrogen peroxide. On the other hand, Strelec et al. [36] found that lime and chestnut honeys exhibited the highest H_2_O_2_ content compared to honeydew, mint, and black locust honeys.

The quantity of H_2_O_2_ mainly depends on the CAT and GOx activities. Therefore, it is expected that the H_2_O_2_ concentration increases when the CAT activity decreases and GOx activity increases. However, this is not always the case [14,43]. In this work, no correlation was found between H_2_O_2_ and GOx and between H_2_O_2_ and CAT (*p* > 0.05) (Table 2). A lack of correlation between GOx and H_2_O_2_ was already reported, concluding that GOX activity is not a reliable parameter for the prediction of H_2_O_2_ and the antimicrobial activity of honey [13,15,23,36].

In our study, there were four behavioral tendencies of the analyzed honeys regarding the CAT and GOx activities and H_2_O_2_ concentration, which can be divided into four different cases:

(1) Samples exhibiting the expected behavior, with H_2_O_2_ mainly being produced by the enzymatic pathway: (a) samples with high GOx activity and non-detected or low CAT activity with a medium-to-high H_2_O_2_ concentration (i.e., H1, H4, H6, H7, C); (b) samples with low GOx activity, showing low H_2_O_2_, regardless of the CAT activity (i.e., HD1, HD3, M1, M2, LV2), and (c) samples with high GOx activity and medium CAT activity with a medium-to-high H_2_O_2_ concentration (H2, H5, HD2, HD5).
(2) Samples showing a lack of statistical correlation between GOx and H_2_O_2_ [23]: samples with high GOx and high CAT with medium-to-low H_2_O_2_ concentration (i.e., H8, HD4, M3). In these samples, although GOx would be able to produce H_2_O_2_, there is high CAT activity that breaks down H_2_O_2_, and therefore the catalase activity neutralizes H_2_O_2_.
(3) Samples in which other factors could be responsible for H_2_O_2_ decomposition, such as metal-containing enzymes (i.e., superoxide dismutase), Fenton reaction in the presence of transition metals, ascorbic acid (can be oxidized by H_2_O_2_ to dehydroascorbic acid), light, and heat [17,42]: samples with medium-to-high GOx activity and non-detected or low CAT with a low H_2_O_2_ concentration (i.e., H3, H10, HB).
(4) Samples showing medium-to-low GOx activity and high H_2_O_2_ concentration (H9, M4, LV1, and HH). In these samples, despite GOx being low, the GOX activity was enough to produce H_2_O_2_, there being a lack of or low CAT activity, as well as other pathways responsible for H_2_O_2_ decomposition, so that it is likely that H_2_O_2_ was kept for longer. Likewise, H_2_O_2_ could also be generated by other pathways, such as polyphenols, that in the presence of transition metals are involved in the generation of H_2_O_2_, through REDOX processes experienced by nectar, as well as by various fungi and yeasts such as *Aspergillus* sp. and *Penicillum* sp., *Saccharomyces* sp. [15,17,20,44].

Therefore, with regard to the H_2_O_2_ contents, significant differences were found among honeys of the same botanical origin, and no differences were found among honeys of different origin. Our results underline the fact that honeys’ H_2_O_2_ concentration does not depend on the botanical origin, being instead affected by the previously described factors.

### 3.3. Antimicrobial Activity

**Figure 2:**
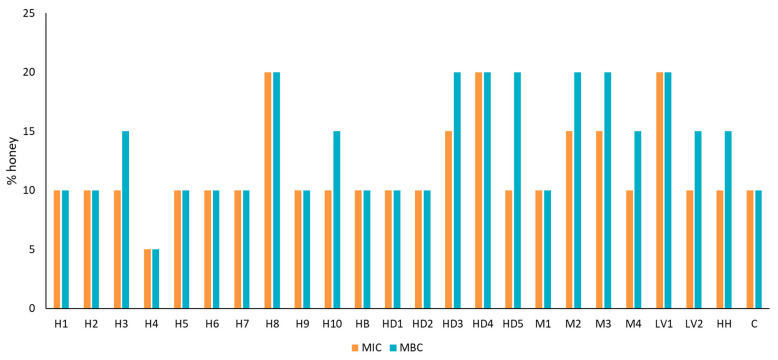
Minimal inhibition concentration (MIC, %, *w*/*v*) and minimal bactericidal concentration (MBC, %, *w*/*v*) of Spanish honeys from different botanical origins against *S. aureus*. H: heather; HB: heather and broom; HD: honeydew; M: multifloral; LV: lavender; HH: holly; C: cornflower. (*n* = 3). Triplicates show identical MBC for each sample. Different MBC values indicate significant differences (*p* < 0.05) among samples.

The MIC and MBC of honey against *S. aureus* ranged from 5% to 20% (Figure 2), highlighting the sample H4 for its higher antimicrobial activity. These values are in accordance with the results obtained by other authors [23,40,44,45] who found MIC values ranging from 2 to 25%, while other researchers found higher MIC values (between 3 and 50%) [15,39]. Manuka honey assayed by Bucekova [44] showed a 5% MIC value and 10% MBC, similar to MIC for H4 and several MBCs of our honeys. The profile of the MBC values was similar or slightly higher than the MIC values, presenting a correlation value of r = 0.7193 (*p* < 0.001) between the MIC and the MBC.

The botanical origins seemed not to be relevant, and no significant correlation was found between the MIC or MBC and H_2_O_2_ or GOx activity. However, heather honeys showed higher GOx and antimicrobial activities, whereas honeydew, multifloral, and lavender honeys showed lower antimicrobial activity than the others, exhibiting lavender honeys with low GOx activities. Therefore, a weak relationship (not statistically significant) was observed between the antimicrobial activity of honey and GOx activity. Other authors found no correlation between antimicrobial activity and H_2_O_2_ nor GOx in honeydew honeys, which they related with the presence of catalase, polyphenols, or natural inhibitors of GOx [15,44,46]. Most researchers found correlations between MIC and H_2_O_2_ concentrations [13,39,40] and between MIC and GOx activities [45], concluding that the antimicrobial properties of honey depend on the accumulation of H_2_O_2_.

Other researchers described higher antimicrobial activity in honeydew honeys than in blossom honeys [13,44], relating dark honeys with higher polyphenol compounds and higher biological activities [47]. Heather honeys are dark honeys, and in previous research, they showed higher phenolics contents than honeydew honeys [48], so it is likely that those compounds contribute to antimicrobial activity.

The sample with the highest antimicrobial activity was H4, which also exhibited a high GOx activity and non-detected CAT, while the six samples that showed MIC values higher than 10% (H8, HD3, HD4, M2, M3, and LV1) and therefore lower antimicrobial activity could have enhanced CAT activity (H8, HD3, HD4, and M3). This fact was confirmed after observing the strong and medium correlations between MIC and MBC and CAT activity (r = 0.6411 and r = 0.4368, respectively) (Table 2). This indicates that the greater the activity of CAT, the greater the MIC and MBC, demonstrating that there is a relationship between antimicrobial activity and the amount of H_2_O_2_ that this enzyme can decompose. A previous correlation was shown by Huidobro et al. [30], who described the activity of this enzyme as the main responsible factor for H_2_O_2_ decomposition and a possible marker of antimicrobial activity of honeys.

**Figure 3:**
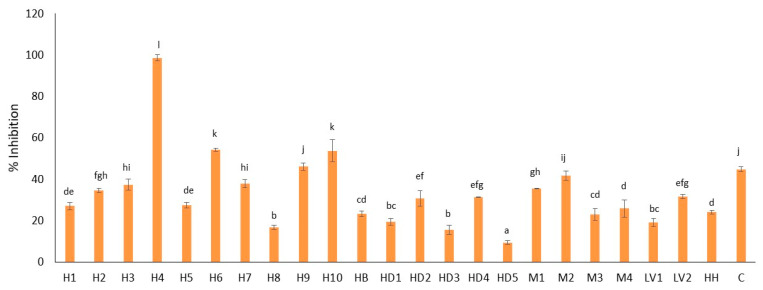
Inhibition percentage (%) of *S. aureus* growth of 5% (*w*/*v*) Spanish honeys from different botanical origin. H: heather; HB: heather and broom; HD: honeydew; M: multifloral; LV: lavender; HH: holly; C: cornflower. a–l: different letters indicate significant differences (*p* < 0.05) between honey samples (*n* = 3).

Despite showing all the honeys with medium-to-high antimicrobial activity, with MBC being lower than or equal to 20% (*w*/*v*), the inhibition percentages obtained for each honey at a concentration of 5% covered a range from 9.43 (HD5) to 98.58% (H4) (Figure 3). Large variations were observed between honeys of the same botanical origin. Other studies [49] presented inhibition percentages against *S. aureus* between 66 and 95% for a 10% honey concentration, so that all the honeys analyzed in this study had greater antimicrobial capacity. In the study by Mirzaei et al. [50], lower inhibition percentages were also obtained at higher concentrations of honey (18–40% at a concentration of 25% honey), which corroborates this fact.

### 3.4. Modeling the Antimicrobial Activity by Means Multiple Regression Models

Linear regression models, using ordinary least squares, have been fitted to explain the antimicrobial activity from CAT and GOx activities and the H_2_O_2_ concentration.

As the MIC and MBC response variables take a small number of different values and, in addition, are observational measures, the percentage of inhibition, %Inh. (5%), has been considered to be more suitable to be modeled.

The model for % Inh. (5%) (Equation (1)) accounts for 75.45% of the variability in the percentage of inhibition. The remaining 24.55% is attributable to deviations from the model, which might be partially due to other factors not included in the study, such as acidity, phenolic compounds, or volatile compounds, among others.

%Inh. (5%) = −0.261892 × AT + 0.0416462 × GOx + 0.420271 × H_2_O_2_ (1)

**Figure 4:**
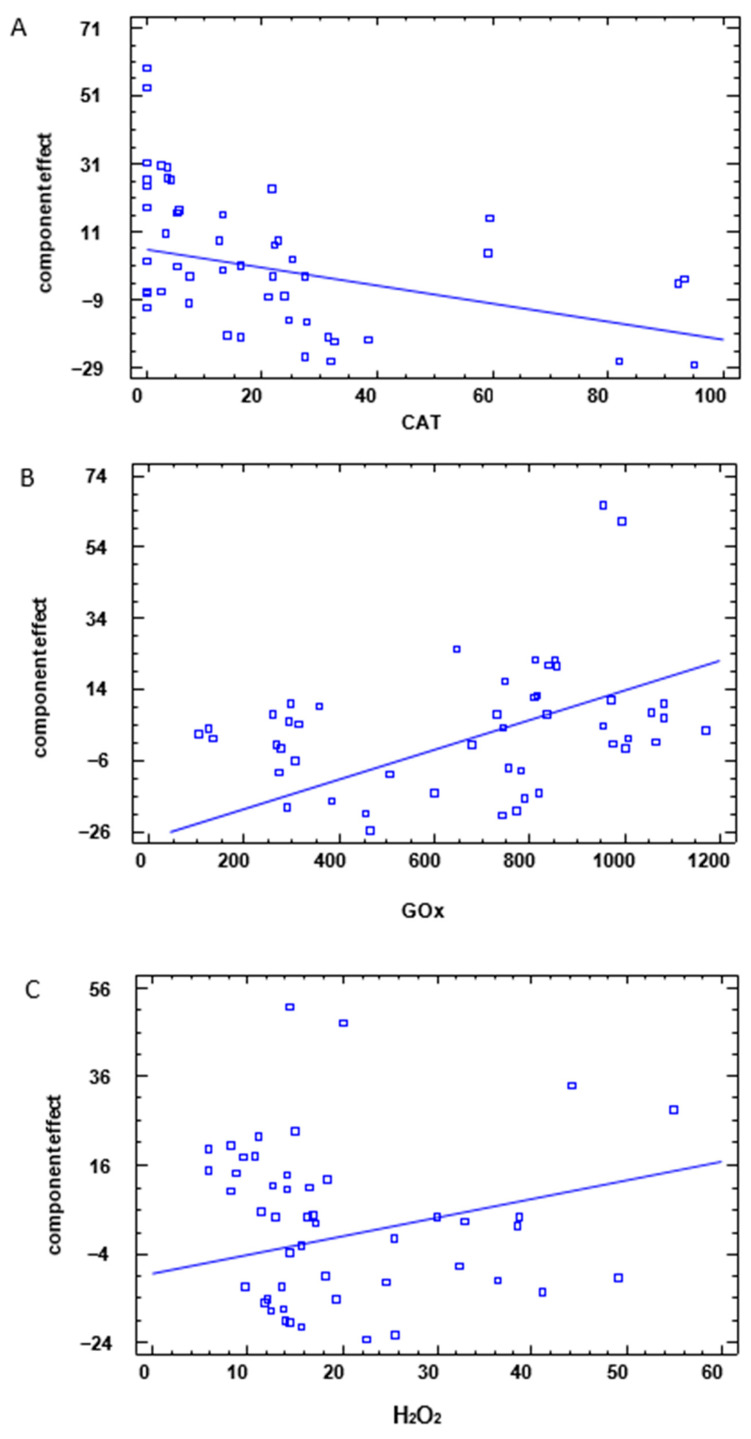
Component effect plots in the %Inh. (5%) model. %Inh. (5%) = −0.261892 × CAT + 0.0416462 × GOx + 0.420271 × H_2_O_2_. (**A**): Component effects plot corresponding to CAT; (**B**): Component effects plot corresponding to GOx; (**C**): Component effects plot corresponding to H_2_O_2_).

To illustrate the effect of each predictor, Figure 4 shows the component effects plot, that is, the portion of the fitted regression model corresponding to each variable. The line on the plot is defined by ${\hat{\beta }}_{j}\left(x_{j}-{\bar{x}}_{j}\right)$, where ${\hat{\beta }}_{j}$ is the estimated regression coefficient for variable j, $x_{j}$ represents the value of variable j as plotted on the horizontal axis, and ${\bar{x}}_{j}$ is its average value. The vertical positions of the points are equal to the component effect plus the residual from the fitted model, which allows us to see the relative importance of a factor compared to the residuals.

In Figure 4A, CAT changes from 0 to 95, while the component effect changes from about 5 to −20, although some residuals are larger than the effect of CAT. Anyway, the first coefficient is negative, meaning that higher levels of CAT lead to lower percentages of inhibitions. Regarding the concentration of GOx, Figure 4B shows that this factor has a positive effect on the percentage of inhibition, given that it changes from about 100 to 1200, whereas the component effect changes from about −26 to 24. Some residuals are also in this case larger than the effect of GOx. Similarly, for a concentration of H_2_O_2_ ranging from about 5 to 55, the component effect changes from −9 to 16 (Figure 4C), although some residuals are larger than the effect of H_2_O_2_, indicating that other important regressors may be missing from the model.

## 4. Conclusions

A relationship was found between catalase activity and antimicrobial activity, so that this enzyme could be successfully used as marker for the antimicrobial activity of honey. Moreover, a linear regression model explained the antimicrobial activity (%Inh. (5%)) from CAT and GOx activities and the H_2_O_2_ concentration.

Spanish honeys showed good antimicrobial activity against *S. aureus*, regardless of their botanical origins. Various factors contributed to this activity, with the generation of H_2_O_2_ being the primary factor. Additionally, several components, such as flavonoids and other polyphenols, likely act synergistically alongside catalase activity. Spanish honeys showed MIC values comparable to those of other medical grade honeys. Therefore, Spanish honeys could be successfully employed for other uses apart from food, improving the potential biological activities of honey-based formulations (i.e., gels, dressing, ointment, cream, paste, syrup, and pastilles), which can help boost local beekeeping. Further research is needed to verify the activity of all peroxide-related parameters during storage, as well as to identify the honey constituents responsible for the strong non-peroxide antibacterial activity of Spanish honeys.

## Figures and Tables

**Table 1:** Botanical origins of honey samples.

Honey	Botanical Origin	Scientific Name
H1	Ling heather	*Calluna vulgaris*
H2	Ling heather	*Calluna vulgaris*
H3	Ling heather	*Calluna vulgaris*
H4	Ling heather	*Calluna vulgaris*
H5	Ling heather	*Calluna vulgaris*
H6	Ling heather	*Calluna vulgaris*
H7	Ling heather	*Calluna vulgaris*
H8	Ling heather	*Calluna vulgaris*
H9	Ling heather	*Calluna vulgaris*
H10	Ling heather	*Calluna vulgaris*
HB	Ling heather and Broom	*Calluna vulgaris*, *Retama* sp.
HD1	Honeydew	
HD2	Honeydew	
HD3	Honeydew	
HD4	Honeydew	
HD5	Honeydew	
M1	Multifloral	
M2	Multifloral	
M3	Multifloral	
M4	Multifloral	
LV1	Lavender	*Lavandula* sp.
LV2	Lavender	*Lavandula* sp.
HH	Holly	*Ilex aquifolium*
C	Cornflower	*Centaurea cyanus*

**Table 2:** Pearson rank correlation coefficients between catalase, glucose oxidase, H_2_O_2_ concentration, MIC, MBC, and % of *S. aureus* inhibition at 5% of Spanish honeys from different botanical origin.

	CAT	GOx	H_2_O_2_	MIC	MBC	% Inh. (5%)
**CAT**						
**GOx**	0.2894 *					
**H_2_O_2_**	−0.2175	−0.1157				
**MIC**	0.6411 ***	−0.0086	−0.0919			
**MBC**	0.4368 **	−0.1252	−0.1480	0.7193 ***		
**% Inh. (5%)**	−0.3784 **	0.1733	−0.0298	−0.4841 ***	−0.5125 ***	

* *p*-value < 0.05; ** *p*-value < 0.01; *** *p*-value < 0.001.

## Data Availability

The data presented in this study are available on request from the corresponding authors. The data are not publicly available due to privacy restrictions.
